# Supplementary figures and images for: Escherichia coli alcohol dehydrogenase YahK is a protein that binds both iron and zinc
Source: PeerJ. 2024 Sep 10;12:e18040. doi: 10.7717/peerj.18040 (PMC11397123; doi:10.7717/peerj.18040)

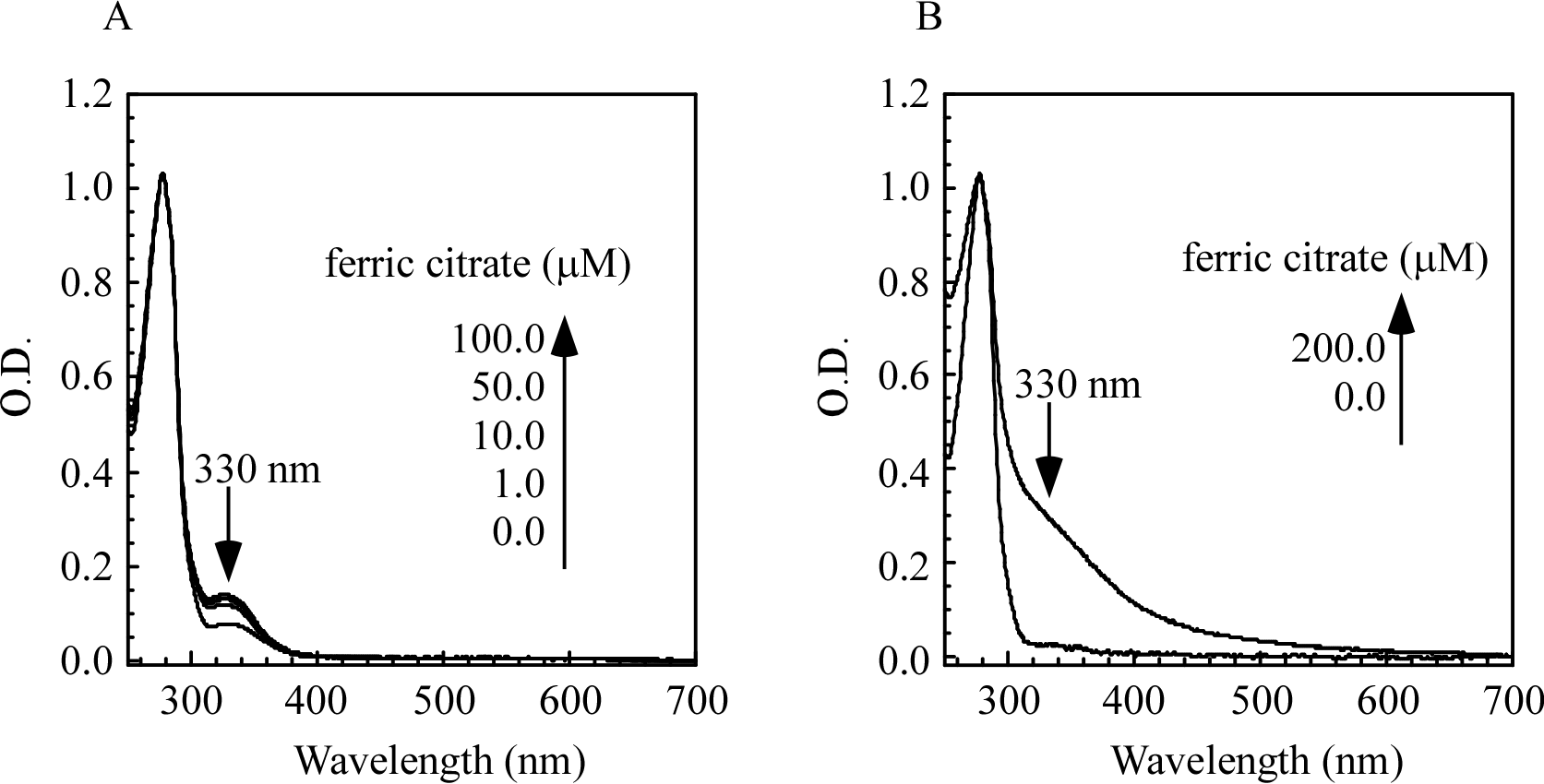

Supplement: Supplemental Information 1 [file peerj-12-18040-s001.png]

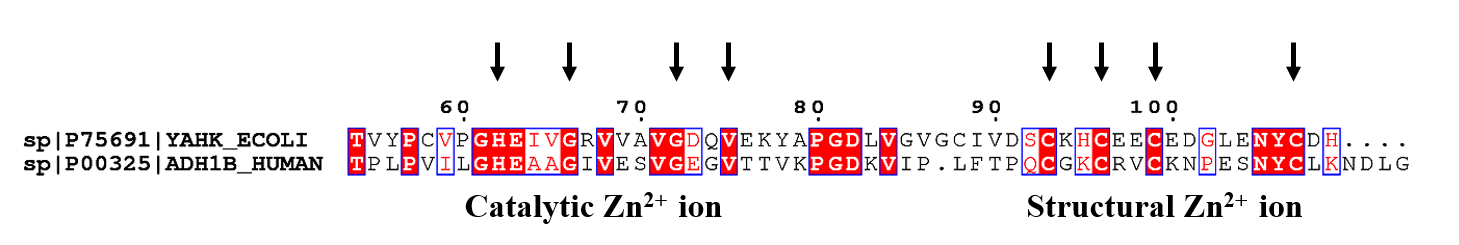

Supplement: Supplemental Information 2 [file peerj-12-18040-s002.png]

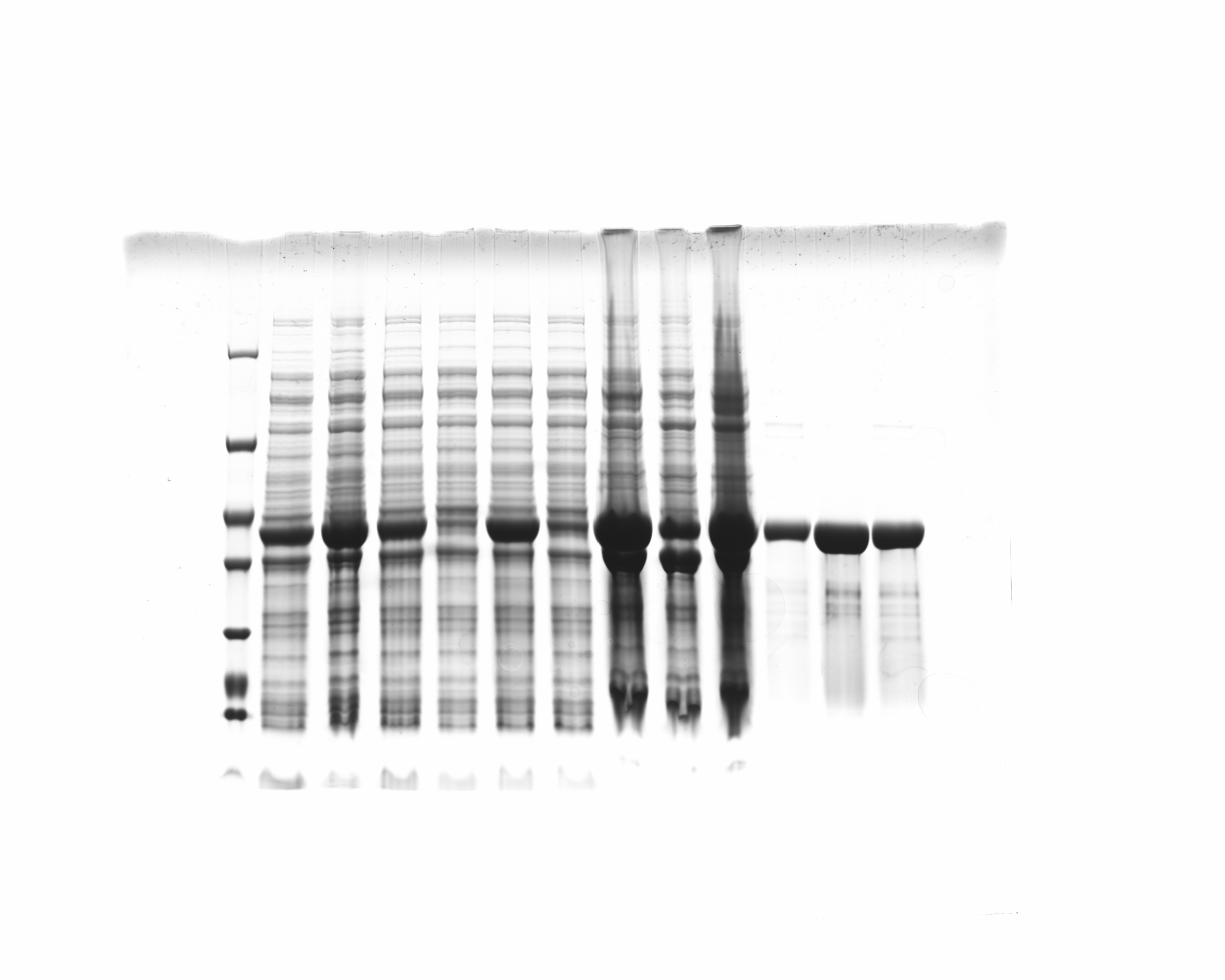

Supplement: Supplemental Information 3 [file peerj-12-18040-s003.zip › Tan 2022-04-12 14h23m56s.tif]
